# A new *Parazuphium* Jeannel, 1942 species (Coleoptera, Carabidae) from the Zagros Mountains in Iran

**DOI:** 10.3897/zookeys.1011.59449

**Published:** 2021-01-18

**Authors:** David W. Wrase, Thorsten Assmann

**Affiliations:** 1 Oderstraße 2, D-15306, Gusow-Platkow, Germany unaffiliated Gusow-Platkow Germany; 2 Institute of Ecology, Leuphana University Lüneburg, Universitätsallee 1, D-21331, Lüneburg, Germany Leuphana University Lüneburg Lüneburg Germany

**Keywords:** Identification key, microphthalmic species, subalpine habitats, Zuphiini

## Abstract

*Parazuphium weigeli***sp. nov.** is described from the Zagros Mountains in Iran. The microphthalmic species was found in a subalpine site under a deeply embedded stone close to a snow field. It resembles *P. salmoni* Assmann, Renan & Wrase, 2015, but differs by shape of pronotum and its punctation, eye size, body proportions, and shape of median lobe of aedeagus and preputial sclerites. An identification key to the known species from Iran is given.

## Introduction

The ground beetle fauna of Iran was compiled by Azadbakhsh and Nozari (2015) in catalogue form. However, soon after the publication of this work, it became clear that the fauna of this large country with its numerous climatic zones and diverse habitats has not been sufficiently studied. Several ground beetle species have since been described (e.g., Azadbakhsh and Wrase 2016; Wrase and Schnitter 2017; Casale and Wrase 2018; Muilwijk et al. 2018; Muilwijk et al. 2019).

We compared two specimens of *Parazuphium* collected from the Zagros Mountains with the other known *Parazuphium* species from the Palaearctic, but it was clear they represented a new species. The description of this new species is given below, and we offer an identification key of the species known from Iran to support further work on ground beetles in this country.

## Material and methods

The material examined is housed in the collections listed below:

**NKME**Naturkundemuseum Erfurt, Germany;

**cWR**Working collection David W. Wrase, Gusow-Platkow, Germany (part of Zoologische Staatssammlung München, Germany).

Dissections were made using standard techniques; genitalia were preserved in “Lompe solution” on acetate labels (Lompe 1989) or in Euparal, and pinned beneath the specimens from which they had been removed.

The following measurements were used:

**BL** Body length: maximal linear distance from the tip of the mandibles to the apex of the right elytron;

**HW** Head width: maximal linear distance;

**A1L–A4L** Length of the antennomeres: from the basal excision to the tip of the given segment (A1L, A2L, A3L, and A4L refer to the length of the antennomeres I (scapus), II, III and IV, respectively);

**PL** Pronotum length: from the anterior to the posterior margin along the midline;

**EL** Elytra length: maximal linear distance from the end of the scutellum to the apex of the right elytron;

**PW** Pronotum width: greatest linear transverse distance across the pronotum;

**EW** Elytra width: maximum distance across the elytra;

**PEW** Prebasal excision width: shortest distance between the two outer pronotal margins;

**PBaW** Pronotal base width: width between the tips of the hind angles at the insertion of the seta.

These measurements were made at magnifications of × 25 and × 50, using an ocular micrometre in a Leica MZ 16 stereo binocular microscope. Microsculpture was examined at a magnification of × 100.

The photos were prepared with an Olympus E-330 digital camera in combination with a Leica MZ 95 stereo binocular microscope. Stacking (up to ~ 100 layers) was done with the software Picolay (www.picolay.de). Drawings were made with a drawing tube attached to the Leica MZ 95.

Labels of type specimens are cited as originally given, and different lines are separated by a forward slash (/).

Types are deposited in Naturkundemuseum Erfurt, Germany (**NKME**) and collections of David W. Wrase in Zoologische Staatssammlung München (**cWR**).

## Results

### Parazuphium weigeli sp. nov.

Taxon classificationAnimaliaColeopteraCarabidae

D8870D70-2769-5E25-886F-2841BFA5ABA5

http://zoobank.org/69A63791-3CDF-4CCF-A0AE-B359EC350BF7

#### Types.

***Holotype*** male: “Iran Zagros Mts., P. Chahar / Mahall va Bachtiari, Asad / Abad 5 km SW, 32°20'30"N, 50°32'59"E, snow field, high pasture, 19.IV.2018, 2500- / 2770 m, leg. A.Weigel” (NKME; left and right antennomeres X and XI are missing). Paratype: 1 female, same data as HT (cWR).

#### Diagnosis.

**Figure 1.**
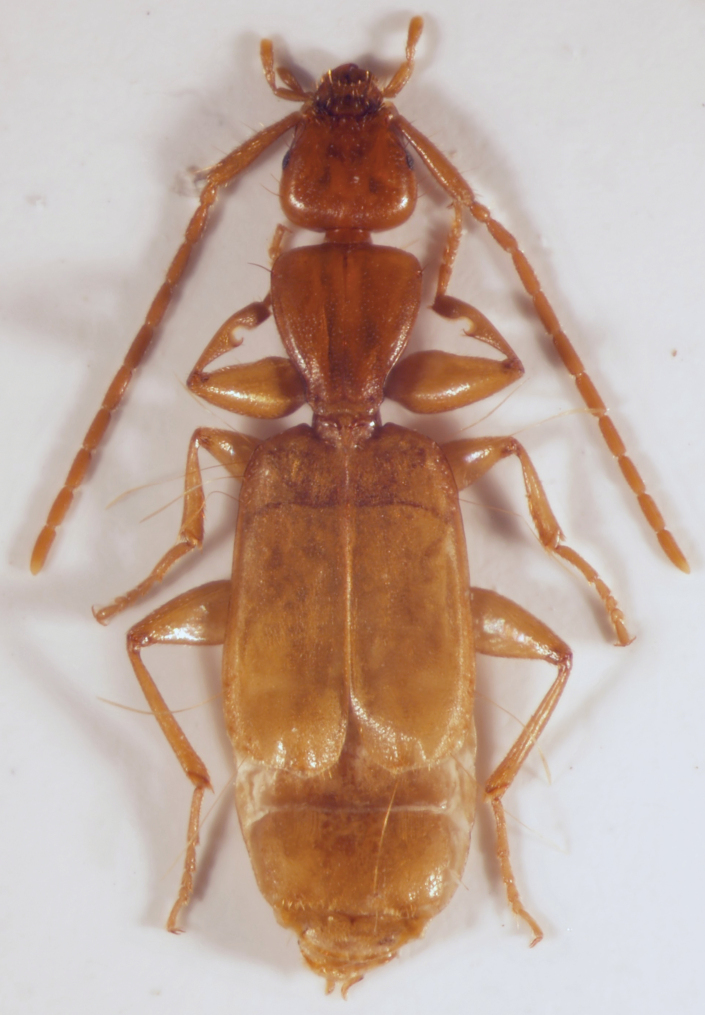
Habitus of *Parazuphium weigeli* sp. nov., paratype, female.

A microphthalmic, depigmented, brachypterous *Parazuphium* species with short, robust legs and moderately long antennae. Median lobe of aedeagus with three sclerites. For habitus see Fig. 1.

#### Description.

BL 3.7–3.9 mm; EW 1.3–1.4 mm.

***Colour***: Light yellowish to brownish.

**Figure 2.**
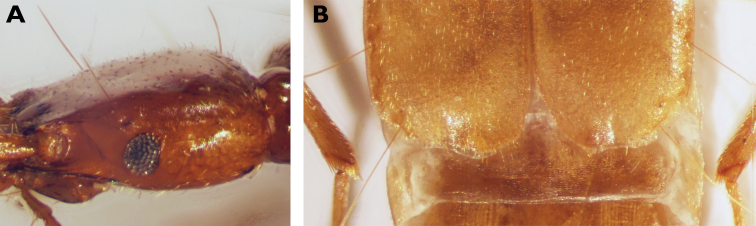
*Parazuphium weigeli* sp. nov. **A** head in lateral view (holotype) **B** apex of elytra (paratype).

***Head*** large, slightly triangular, with rounded tempora. Eyes small and flat, ~ 1/3 as long as tempora (dorsal view) (Figs 1, 2a). Antennae moderately long, scapus (antennomere I) somewhat shorter than width of head (A1L/HW 0.82–0.83), ~ 5 × as long as antennomere II (A1L/A2L 4.7–5.17), antennomere II somewhat longer than 1/2 antennomere III (A2L/A3L 0.54–0.58), antennomere III somewhat shorter than antennomere IV (A3L/A4L 0.92). Neck somewhat wider than 1/3 of head width. Surface moderately shiny, punctures somewhat indistinct and scattered, setae also fine and scattered. Microsculpture consisting of isodiametric meshes.

***Pronotum*** somewhat wider than head (HW/PW 0.88–0.91), slightly longer than wide (PW/PL 0.92), widest at approximately the apical 1/6, where lateral seta is inserted. Anterior margin almost rectilinear, somewhat excavated at insertion of neck. Anterior angles rounded, but hardly prominent. Lateral margin in apical 2/3 continuously rounded, concavely curved at the prebasal excision. Posterior angles obtuse and weakly prominent (PBaW/PEW 1.02–1.05), distinctly shifted forward due to a strong excision of the basal margin (Fig. 4A). Disc flat, median line medially somewhat excavated. Median sulcus fine, impressed on the posterior margin, not reaching the basal margin. Regularly and more strongly punctate and setose than head, microsculpture with irregular transverse meshes.

***Elytra*** short (EL/EW 1.28–1.31), subparallel, distinctly widening to the apex. Apical margin sinuous (Fig. 2b). Striae weak, only intimated, somewhat irregular, internal intervals weakly convex. Punctuation denser than on pronotum, somewhat wrinkled. Surface with setosity formed by short fine setae (somewhat denser than on pronotum), inclined backwards, setae at apex somewhat longer. Series umbilicata consists of eight (five long and three short) humeral and five (three long and two short) apical setae. Surface moderately shiny, microsculpture with irregular meshes. Brachypterous.

***Legs*** robust and short, protibia somewhat curved inwards, mesotibia bent slightly outwards, metatibia straight. Male protarsomeres I–IV enlarged and with adhesive setae beneath.

**Figure 3.**
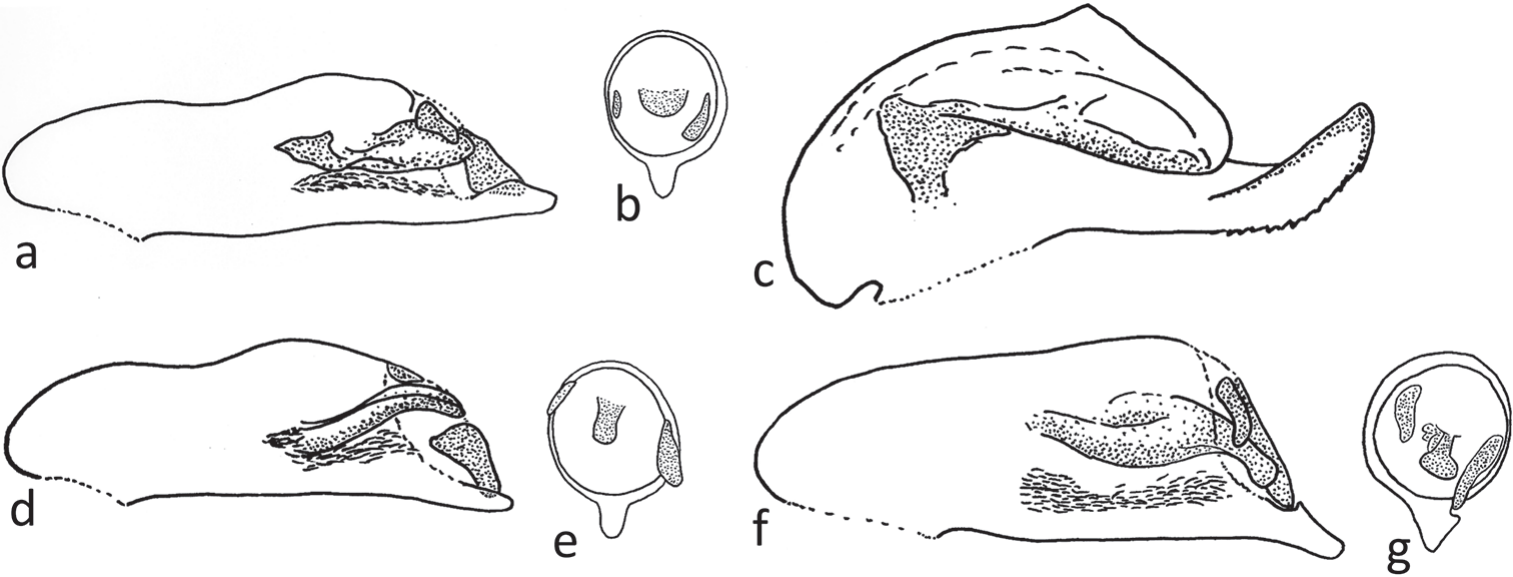
Median lobe of aedeagus of *Parazuphium***a, c, d, f** lateral views **b, e, g** view onto oroficium (preputial field) **a, b***P. weigeli* sp. nov. **c***P. damascenum***d, e***P. salmoni***f, g***P. chevrolatii*.

***Median lobe of aedeagus*** ~ 0.75 mm long, strongly sclerotised. Dorsal side sinuous, apex pointed. Preputial field with three sclerites; the central one large, prolonged, at the tip spoon-like rounded, but central and basal part irregularly shaped; the left one rudimentary; the right one triangular (Fig. 3a, b). Left paramere rounded, larger than the strongly reduced right one.

#### Comparisons.

The new species belongs to the genus *Parazuphium* because of small, numerous, erect setae on the scapus, one pair of supraorbital setae, a sinuous apical margin of the elytra, and a tube-shaped median lobe of the aedeagus with small sclerites on the preputial field.

**Figure 4.**
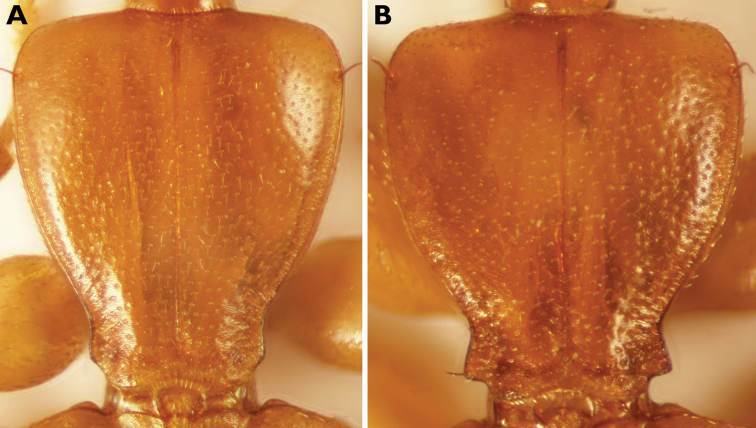
Pronotum of *Parazuphium***A***P. weigeli* sp. nov. **B***P. salmoni*.

**Figure 5.**
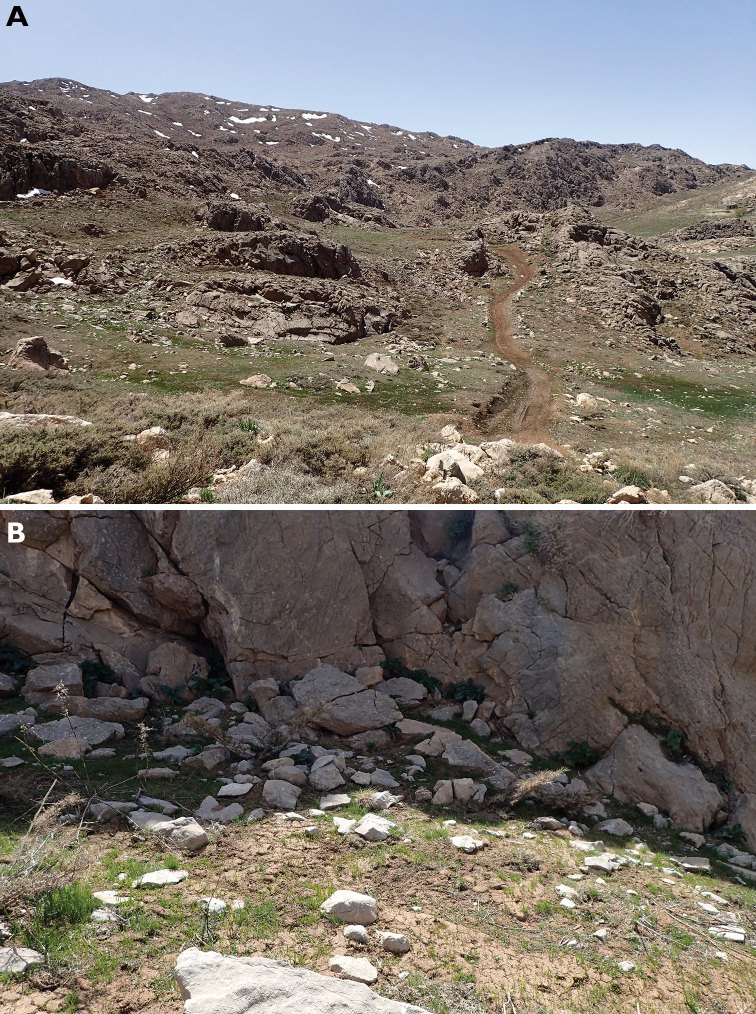
Habitat of *Parazuphium weigeli* sp. nov. **A** landscape **B** microhabitat with the stone under which the two individuals were found. Photographs by Andreas Weigel.

In its characters it is similar to *P. salmoni* Assmann, Renan & Wrase, 2015. It shares with it the small eyes, strong and relatively short legs, moderately long antennae, metatibia straight in both sexes, similar body indices, brachyptery, and the colour. It differs from *P. salmoni* by the following features: (1) eyes larger (eye diameter in dorsal view ~ 1/3 of temple length, in *P. salmoni* ~ 1/4); (2) antennomere I shorter in relation to antennomere II, ~ 5 × as long as antennomere II (in *P. salmoni* 5.5–6×) (measured from the basal incision of the antennomeres to the apex); (3) pronotum more slender in relation to head (HW/PW 0.88–0.91, in *P. salmoni* 0.83–0.85); (4) hind angles less prominent (Fig. 4); (5) punctuation of pronotum less prominent (Fig. 4); (6) microsculpture of pronotum weaker and transverse (in *P. salmoni* isodiametric); (7) median lobe of aedeagus not downwardly bent and with different configuration and shape of the sclerites of the preputial field (Fig. 3a, b vs. Fig. 3d, e).

The new species differs from *P. damascenum* (Fairmaire, 1897) by (1) straight metatibia in both genders; (2) smaller and flat eyes; (3) brachyptery, (4) deviating shape of median lobe of aedeagus and its sclerites (Fig. 3a–c).

*Parazuphium weigeli* sp. nov. differs from *P. chevrolatii* by: (1) smaller eyes; (2) antennomere I shorter, shorter than head width; (3) weakly developed elytral intervals; (4) slender median lobe of aedeagus without an excision at the base of prolonged apical tip (Fig. 3a, b vs. Fig. 3f, g).

The other microphthalmic species of the genus (*P. angustioculum* Hůrka, 1982, *P. baeticum* (K. & J. Daniel, 1898), *P. punicum* (K. & J. Daniel, 1898), *P. ramirezi* J. & E. Vives, 1976) differ from *P. weigeli* sp. nov. by their (1) slender legs, (2) longer body lengths, (3) deviating proportions of antennomeres, (4) shape of median lobe of aedeagus and number and shape of sclerites (cf. Hůrka 1982, 1987). These species occur exclusively in the south-western part of the Palaearctic (Morocco, Spain, and Sicily) (Huber and Marggi 2017).

#### Habitat.

Habitat is a subalpine grassland in 2500–2770 m a.s.l. The specimens were found under a large stone, deeply embedded in the soil, close to a snow field (Fig. 5).

#### Dispersal power.

Brachypterous.

#### Distribution.

Known so far only from the type locality. As the species is incapable of flight, its dispersal power is strongly limited, and a wider distribution range outside the Zagros Mountains is unlikely. Further studies may show if the occurrence of the species is restricted to high altitudes.

#### Etymology.

Latinised patronym based on the surname of our colleague and friend, Andreas Weigel (Pößneck, Germany), specialist of Cerambycidae. He collected the first specimens of this new species and contributed to carabidology with numerous records of ground beetles, including new taxa.

### Identification key to the known *Parazuphium* species from Iran

The genus *Parazuphium* can be easily distinguished from the other genera of the tribe by the following features: (1) scapus of antennae with moderately long setae which poke (more or less at a right angle) out of the normal hairs; these setae are shorter than the large apical seta but longer than the regular vertically protruding bristles (Assmann et al. 2015: fig. 5). (2) Median lobe of aedeagus compact and heavily sclerotised, with a small membranous preputial field. (3) Two pairs of supraorbital setae (the posterior pair located in the basal quarter of the head). (4) Apical margin of elytra sinuous. (5) Genital segment almost rectangular, sometimes bilaterally symmetrical, but not rounded, and heavily sclerotised.

**Table:** 

1	Metatibia strongly curved, especially in males (Assmann et al. 2015: fig. 10a, b). Eyes larger, laterally protruding. Median lobe of aedeagus with tip bent upwards and lower side like a saw blade (Fig. 3c). Body length 3.5–5.2 mm	***Parazuphium damascenum* (Fairmaire, 1897)**
–	Metatibia almost straight in both genders (Fig. 1; Assmann et al. 2015: fig. 10c, d). Eyes of variable size (Fig. 2a; Assmann et al. 2015: fig. 11a, b)	**2**
2	Eyes strongly reduced (Fig. 2a), temples at least 3 × as long as eyes (dorsal view). Antennae shorter, scapus shorter than the head width, antennomere II < 2 × as long as wide. Median lobe of aedeagus with different configuration and shape of the sclerites of the preputial field (Fig. 3a, b). Body length 3.7–3.9 mm	***Parazuphium weigeli* sp. nov.**
–	Eyes of variable size (Assmann et al. 2015: fig. 11a, b), but shorter than temples (dorsal view). Antennae elongate, scapus longer than head width, antennomere II ~ 2 × as long as wide. Median lobe of aedeagus with an excision at the base of prolonged apical tip (Fig. 3f, g). Body length 4–6.5 mm	***Parazuphium chevrolatii* Castelnau de Laporte, 1833**

## Supplementary Material

XML Treatment for Parazuphium weigeli
